# Pediatric long COVID: relationships with premorbid history of anxiety or depression and health-related quality of life

**DOI:** 10.1093/jpepsy/jsaf034

**Published:** 2025-05-06

**Authors:** Ellen Henning, Rashelle Musci, Sara B Johnson, Cindy Villatoro, Laura A Malone

**Affiliations:** Department of Behavioral Psychology, Kennedy Krieger Institute, Baltimore, MD, United States; Department of Psychiatry and Behavioral Sciences, Johns Hopkins University School of Medicine, Baltimore, MD, United States; Department of Mental Health, Johns Hopkins University School of Public Health, Baltimore, MD, United States; Department of Mental Health, Johns Hopkins University School of Public Health, Baltimore, MD, United States; Department of Pediatrics, Johns Hopkins University School of Medicine, Baltimore, MD, United States; Center for Movement Studies, Kennedy Krieger Institute, Baltimore, MD, United States; Department of Neurology, Kennedy Krieger Institute, Baltimore, MD, United States; Department of Neurology, Johns Hopkins University School of Medicine, Baltimore, MD, United States

**Keywords:** Long COVID, Pediatrics, Mood, Anxiety, Self-Report

## Abstract

**Objective:**

Up to 25% of youth may develop long COVID following COVID-19 infection. Mood changes are commonly reported; however, few studies use validated measures. This study describes prevalence of self-reported anxious and depressive symptoms among youth with long COVID. We also examined the association of these symptoms with prior mental health diagnosis and health-related quality of life.

**Methods:**

We conducted a retrospective study of pediatric patients (*n* = 139) evaluated in a pediatric post-COVID-19 rehabilitation clinic who met criteria for long COVID. Patients were included if they completed the Pediatric Quality of Life Inventory (PedsQL), the Multidimensional Anxiety Scale for Children, second edition (MASC 2), and/or the Children’s Depression Inventory, second edition (CDI 2). Relationships between prior anxiety or mood disorder and current depressive and anxious symptoms were assessed using chi-square tests. Relationships between depressive and anxious symptoms and health-related quality of life were examined using multiple linear regression.

**Results:**

Almost 40% of patients had elevated scores for anxious or depressive symptoms. Prior anxiety or mood disorder diagnosis was associated with higher scores. Depression scores, and specifically the Ineffectiveness subscale, were inversely associated with PedsQL scores.

**Conclusions:**

Prevalence of anxious and depressive symptoms in this clinical sample was high. Screening measures for mood and anxiety overlap with physical symptoms of long COVID and use of collateral information is recommended. The relationship between the Ineffectiveness subscale and the PedsQL warrants further investigation to evaluate if they assess the same domain or if negative perception of abilities contributes to health-related quality of life.

According to estimates based on seroprevalence by the Centers for Disease Control and Prevention (2022), over 90% of children and adolescents in the United States have had at least one COVID-19 infection. While most individuals who experience a COVID-19 infection recover within a few days to weeks, some individuals may experience ongoing or new symptoms for an extended period of time, which is known as long COVID (Department of Health and Human Services, 2022). By 2022, 1.3% of the pediatric population in the United States was estimated to have had long COVID (Vahratian et al., 2023). Incidence rates across studies since the beginning of the pandemic have varied widely from 4% to 62% for pediatric long COVID, which is likely a reflection of heterogeneity in study design and diagnostic criteria (Rao et al., 2024). Additional systematic reviews have estimated the prevalence of long COVID in children who were infected by SARS-CoV-2 to be between 5% and 25%, depending on the definition of long COVID used (e.g., 4-week duration vs. 3-month duration), and population studied (e.g., hospitalized vs. nonhospitalized) (Lopez-Leon et al., 2022; Zimmerman et al., 2021).

Long COVID is often multisystemic. It is considered an infection-associated chronic condition similar to conditions triggered by infections such as Epstein-Barr, influenza, SARS, West Nile, etc., which often present with similar symptoms (e.g., fatigue, cognitive difficulties, pain, autonomic dysfunction; National Academies of Sciences, Engineering, and Medicine, 2024). Common symptoms of long COVID in children and adolescents include fatigue, cognitive changes (e.g., difficulty with attention or concentration or cognitive fatigue), sleep difficulties, joint and body pain, headaches, gastrointestinal problems, dizziness and autonomic dysfunction, and mood changes (Chen et al., 2023; Lopez-Leon et al., 2022; Malone et al., 2022; Zimmerman et al., 2021). Physical symptoms often have a negative functional impact on such activities as school attendance, social activities, and activities of daily living such as showering and eating (Buonsenso et al., 2023). Unfortunately, in addition to social isolation, youth with long COVID may also experience social stigma. For example, others may not believe that long COVID is a real medical condition, which can impact interpersonal relationships as well as trust in healthcare professionals (Buonsenso et al., 2023).

Regarding mood changes specifically, Lopez-Leon et al. (2022) found that mood symptoms (including increased sadness, tension, anger, anxiety, and depression) were the most common long COVID symptoms reported across pediatric studies (16.5% pooled prevalence). Influences on mood may be multifactorial. Hypothesized mechanisms of pathophysiology have included dysfunction in inflammatory and immune system responses, as well as low peripheral serotonin levels (Fernández-Castañeda et al., 2022; Wong et al., 2023). However, mood symptoms may have been environmentally influenced by psychosocial and sociopolitical stressors from the COVID-19 pandemic as a whole and may reflect the general increase in mental health concerns (Solmi et al., 2022). Also, as mentioned before, long COVID symptoms can influence daily functioning, which may influence mood and overall quality of life (Buonsenso et al., 2023).

To investigate the risk of mental health concerns specifically due to long COVID, Mat Hassan et al. (2023) compared pooled prevalence for mental health concerns in studies of children with and without long COVID. Their analysis indicated 9% of children with long COVID experienced anxiety and 15% experienced depression. The odds of depression and anxiety were each twice times higher in those with long COVID compared to those without long COVID. However, they noted significant heterogeneity in methods for the included studies, with most studies relying on binary yes/no identification of concerns without assessment using validated measures of anxiety and depression (2023).

Very few studies have included assessment of anxious and depressive symptoms using validated measures to understand neuropsychiatric symptoms in long COVID. Scarselli et al. conducted a prospective study using the Multidimensional Anxiety Scale for Children, second edition (MASC 2) and the Child Depression Inventory, second edition (CDI 2) in addition to measures of overall behavior, sleep, and neuropsychological assessment. Results indicated higher than expected neuropsychiatric symptoms compared to world prevalence data. Guido et al. (2022) also utilized the MASC 2 and CDI 2 to evaluate anxious and depressive symptoms, respectively, in the context of evaluating neurological and psychological symptoms of long COVID in children. They found high rates of depressive and anxious symptoms and that anxious and post-traumatic stress symptoms were significantly associated with neurological symptoms of long COVID.

Research in adults with long COVID suggests that a prior psychiatric diagnosis is among the most robust predictors of experiencing psychiatric symptoms following long COVID (Hovagemyan et al., 2023). However, the relationship between a history of mental health diagnosis and anxiety and depressive symptoms in pediatric long COVID has not been established in the literature to date.

Prior studies demonstrate that children with long COVID have significantly poorer self-reported quality of life overall and in multiple domains (i.e., fatigue, family impact) compared to normed samples (Chen et al., 2023). While a premorbid history of depression has been linked to poorer self-reported fatigue quality of life scales (Chen et al., 2023), prior studies have not evaluated the concurrent association between anxiety and depression symptoms and quality of life among children with long COVID using validated measures.

This study aims to describe the prevalence of self-reported anxiety and depressive symptoms in a clinical sample of children and adolescents with long COVID utilizing validated measures. Additionally, we then evaluate the relationship between history of mood and/or anxiety diagnosis and the likelihood of self-reported anxiety and depressive symptoms after a long COVID diagnosis. We hypothesize that prior mental health diagnosis will be associated with greater symptom severity. Finally, we investigate the relationship between anxiety and depressive symptoms and self-reported health-related quality of life at the time of long COVID diagnosis. We hypothesize that anxiety and depressive symptoms after a long COVID diagnosis will each be associated with poorer quality of life.

## Methods

### Procedures

All study procedures were approved as exempt by the Johns Hopkins Medicine Institutional Review Board. Data were collected from patient’s electronic medical records, intake surveys completed by families as part of clinic procedures, and patient self-reported measures of mood, anxiety, and quality of life. All data were housed in a database registry for long COVID.

### Patients

Each patient was seen for an initial appointment in a multidisciplinary pediatric post-COVID-19 rehabilitation clinic. Patients for this study were excluded if they were not diagnosed with long COVID during the clinic visit as defined by the United States Department of Health and Human Services (2023). Notably, the criteria for long COVID at the time of the clinic visit for this cohort of patients were defined as ongoing symptoms following COVID-19 infection present for 4 weeks or more after the initial phase of infection (2023). In 2024, the national definition of long COVID was changed to extend the timeline of symptoms following the initial phase of infection to 3 months after the initial infection (2024). Four patients would not have met the new criteria at the time of the initial visit but have since met criteria at follow-up visits. In other words, they had experienced symptoms for 2 months at the time of the initial visit but continued to have persistent symptoms for 3 months or more at follow-up visits. Patients were also excluded if they did not meet age criteria for the Pediatric Quality of Life Inventory (PedsQL; see description below), which has the broadest age range of all measures completed. Data were collected from November 5, 2020 to April 4, 2024. A total of 146 patients completed the PedsQL during this time frame. Seven patients were excluded for either not meeting criteria for long COVID (*n* = 3) or for not meeting age criteria for the mental health measures (*n* = 4). Data are available on request.

### Measures

#### Demographic data

Demographic data were collected via phone with legal caregivers during the clinic intake process during which a medical record was created. This information was then pulled directly from the medical record for analysis. Additional information regarding COVID-19 infection dates, initial symptoms, hospitalization, current symptoms of concern, medical providers who had completed workup prior to clinic visit related to current symptoms of concern, past medical history, and vaccination status were collected from families via clinic questionnaire prior to or during the initial clinic visit. Past psychiatric history was collected during the clinic visit via clinical interview with the clinical psychologist.

#### Multidimensional Anxiety Scale for Children, second edition

The MASC 2 (March, 2013) self-report version measures anxiety-related symptoms for children and adolescents between 8 and 19 years of age. It provides a total score which represents the range and severity of anxiety symptoms overall. The MASC 2 also has six individual scale scores (i.e., Separation Anxiety/Phobias, Generalized Anxiety Disorder Index, Social Anxiety: Total, Obsessions & Compulsions, Physical Symptoms: Total, and Harm Avoidance) to provide information about which groups of symptoms are most problematic. Patients complete 50 items which are scored using a 4-point Likert scale. Scores are converted to standardized *T* scores which have a mean of 50 and a standard deviation of 10. On this measure, *T* scores below 60 are considered average, scores between 60 and 64 are considered slightly elevated, scores between 65 and 69 are considered elevated, and scores 70 and above are considered very elevated. Thus, we have categorized *T* scores within 1–2 *SD*s from the mean as slightly elevated to elevated and these scores are considered together. The MASC 2 has acceptable internal consistency and strong test–retest reliability. Additionally, it has good discriminant validity and convergent validity with the Beck Youth Inventory-Anxiety measure (2013).

#### Children’s Depression Inventory, second edition

The CDI 2 (Kovacs, 2011) self-report version measures depressive symptoms in children and adolescents between 7 and 17 years of age. The CDI 2 provides a Total score and two scaled scores: Emotional Problems and Functional Problems. Responses on the Emotional Problems scale are focused on negative feelings such as sadness and guilt, anhedonia, and changes in sleep, appetite, and energy levels. This scale is further subdivided into two subscales: Negative Mood/Physical Symptoms and Negative Self-Esteem. The Functional Problems scale provides information about interpersonal difficulties and decreased performance in areas such as school, preferred activities, and peer or family relationships. This scale is also subdivided into two subscales: Ineffectiveness and Interpersonal Problems. The CDI 2 contains 28 items and scores are converted to standardized *T* scores which have a mean of 50 and a standard deviation of 10. On this measure, *T* scores below 60 are considered average, scores between 60 and 64 are considered high average, scores between 65 and 69 are considered elevated, and scores 70 and above are considered very elevated. Thus, we have categorized *T* scores within 1–2 *SD*s from the mean as slightly elevated to elevated and these scores are considered together. For each item, patients are provided with three statements which translate to a 3-point Likert Scale. It has good discriminant validity and appropriately differentiates individuals with depressive symptoms from youth without them (2013).

#### The Pediatric Quality of Life Inventory

The Pediatric Quality of Life Inventory (PedsQL) (Varni et al., 2001) generic core scale self-report version measures health-related quality of life in children and adolescents with or without health concerns. The self-report measure can be administered to children ages 5 years to 7 years as an interview and from 8 years to 25 years as a questionnaire. It includes 23 items which provide a total scale summary score and four scale scores: physical functioning, emotional functioning, social functioning, and school functioning. Items are scored on a 5-point Likert scale with higher scores indicating better quality of life. In prior publications (Huang et al., 2009), cutoffs for the PedsQL total score have been defined utilizing Clinical Risk Groups software to identify children with special healthcare needs or chronic conditions. Their defined cutoffs are classified as 78 for “mild chronic health condition” (comparable to scores of patients with attention-deficit/hyperactivity disorder per Huang et al.), 76 for “moderate chronic health condition” (e.g., comparable to scores of patients with asthma per Huang et al.), and 70 for “major chronic health condition” (e.g., comparable to scores of patients with cystic fibrosis or cancer per Huang et al.) for children 8 years of age and older. The PedsQL has been demonstrated to have good validity in distinguishing between children with health concerns and without health concerns and disease severity within a chronic health condition and has good reliability (Varni et al., 2001).

### Statistical analysis

Data exploration and descriptive statistics were conducted using the Psych Package in R (Revelle, 2024). Means and standard deviations were generated for MASC, CDI 2, and PedsQL total scores and their respective subscales. Chi-square tests were used to evaluate the relationship between a pre-existing history of anxiety or mood disorder (present/absent) and depression and anxiety score categories (i.e., average, slightly elevated/elevated, very elevated). Multiple linear regression was used to evaluate the relationship between mental health and quality of life. As part of this analysis, adjusted regression estimates (est.), and *p*-values were generated using individual patient-level data while accounting for patient’s sex assigned at birth and age due to age and sex being associated with prevalence of long COVID and symptom presentation (Rao et al., 2024). This analysis was done utilizing the base R software (R Core Team, 2021).

## Results

One hundred and thirty-nine patients were included in the analysis based on completion of the PedsQL and other inclusion criteria. Demographic data regarding sex assigned at birth, age, race, time since COVID-19 infection, hospitalization rates, vaccination status, and school status are presented in Table 1.

**Table 1.** Demographic information for participants (*n* = 139).

Sex assigned at birth	
Female	61%
Male	39%
Age	
Mean	14 years
Range	5–22 years
Race (per medical record designation)	
White	72%
Black	10%
Multiracial	5%
Asian	3%
Hispanic or Latinx	3%
Other	1%
Unknown/not listed	6%
Time since COVID-19 infection	
Mean	12 months
Range	2–35 months
COVID-19 vaccination status	
At least 1 vaccine	48%
None	52%
Hospitalization from COVID-19	
Yes	10%
No	90%
School status	
Full-time in person or homeschooled unrelated to physical symptoms	54%
Attending partial day	15%
Full-time homebound instruction or homeschooled related to physical symptoms	23%
Withdrew from school/not attending	7%
Graduated high school and not in school	1%
Reported current concerns with school performance	
Yes	85%
No	15%
Premorbid mood or anxiety disorder	
None	51%
Mood disorder	2%
Anxiety disorder	28%
Both mood and anxiety disorder	19%

The descriptive statistics for the PedsQL, MASC 2, and CDI 2 are presented in Table 2. For MASC 2 Index scores, the following percentages were derived using *T* scores in the elevated or very elevated range (i.e., 65+): 27% for Separation Anxiety/Phobias, 50% for Generalized Anxiety Disorder, 21% for Social Anxiety, 25% for Obsessions & Compulsions, 57% for Physical Symptoms, and 4% for Harm Avoidance. For the CDI 2, 36% had elevated scores for the Emotional Problems scale, with 44% elevated scores on the Negative Mood/Physical Symptoms subscale and 21% elevated scores on the Negative Self-Esteem subscale. For the Functional Problems scale, 30% had elevated scores, with 34% elevated scores for the Ineffectiveness subscale and 24% elevated scores on the Interpersonal Problems subscale.

**Table 2.** Descriptive statistics for PedsQL, MASC 2, and CDI 2.

Measure/scale	*N*	*M*	Range	*SD*
PedsQL				
Total score	139	55.00	0–94.57	17.68
Physical functioning	139	49.02	0–100	24.97
Emotional functioning	139	56.69	0–100	21.71
Social functioning	139	73.43	0–100	20.43
School functioning	139	44.61	0–100	21.79
MASC 2				
Total score	112	61.01	40–90	13.34
Separation anxiety/phobias	112	56.27	40–90	13.00
Generalized anxiety disorder	112	63.75	40–90	13.18
Social anxiety	112	54.91	40–81	12.35
Obsessions and compulsions	112	56.32	40–90	12.49
Physical symptoms	112	65.69	40–90	12.76
Harm avoidance	112	52.34	40–69	7.72
CDI 2				
Total score	101	61.18	40–90	12.92
Emotional problems	101	60.92	41–90	12.05
Negative mood/physical symptoms	101	62.92	41–90	12.05
Negative self-esteem	101	54.67	43–90	12.36
Functional problems	101	59.46	40–90	13.74
Ineffectiveness	101	53.90	40–90	13.33
Interpersonal problems	101	53.90	41–90	13.68

*Note.* PedsQL = Pediatric Quality of Life Inventory; MASC 2 = Multidimensional Anxiety Scale for Children, second edition; CDI 2 = Children’s Depression Inventory, second edition.

**Figure 1.**
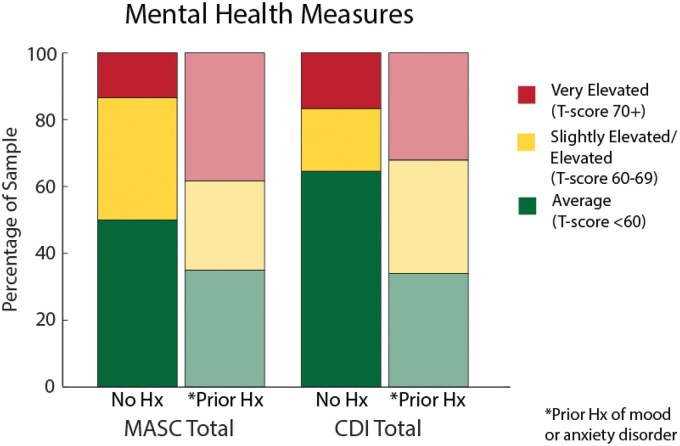
Score distribution for the MASC 2 and CDI 2 total scores based on pre-existing history of anxiety or mood disorder. *Note*. MASC 2 = Multidimensional Anxiety Scale for Children, second edition; CDI 2 = Children’s Depression Inventory, second edition.

Figure 1 illustrates the distribution of scores for the MASC 2 and CDI 2 for this sample based on our categorization of the three groups (average, slightly elevated/elevated, very elevated) and whether or not there was a premorbid mood or anxiety diagnosis. For the MASC 2, 42% of patients had average scores, 31% had slightly elevated/elevated scores, and 27% had very elevated scores. For the CDI 2, 49% of patients had average scores, 26% had slightly elevated/elevated scores, and 26% had very elevated scores. Patients that had a pre-existing history of anxiety or depression were more likely to have scores in the elevated to very elevated categories on the CDI 2, χ^2^ = 9.47, *p* = .01, and on the MASC 2, χ^2^ = 8.80, *p* = .01. However, there were also many children and adolescents with long COVID in our sample without a premorbid history of mental health conditions that scored in the slightly elevated to elevated and very elevated categories for total anxiety score on the MASC 2 (23% in elevated range or above) and total depression score on the CDI 2 (17% in elevated range or above).

We next investigated whether MASC 2 or CDI 2 standardized scores were related to health-related quality of life (PedsQL) mean score, accounting for age and sex. All regression results are provided in Table 3. Only the CDI 2 total score was significantly associated with PedsQL mean score (est. = −0.63, *p* < .001). MASC 2 total score (est. = −0.28, *p* = .09), female sex (est. = 1.88, *p* = .56), and age (est. = −1.07, *p* = .09) were not significantly associated with quality of life.

**Table 3.** Regression analyses.

Model 1: MASC 2, CDI 2, age, and sex with PedsQL total score			
Term	Estimate	*SE*	*p*
Intercept	124.63	10.25	<.001
CDI 2	−0.63	0.17	<.001
MASC 2	−0.28	0.17	.09
Sex (female)	1.88	3.21	.56
Age	−1.07	0.62	.09

Model 2: Emotional problems, functional problems, MASC 2, age, and sex with PedsQL total score			
Term	Estimate	*SE*	*p*
Intercept	125.86	10.42	<.001
CDI 2 emotional problems	−0.15	0.22	.50
CDI 2 functional problems	−0.54	0.19	.006
MASC	−0.24	0.17	.17
Sex (female)	2.13	3.28	.52
Age	−1.20	0.63	.06

Model 3: Interpersonal problems, ineffectiveness, MASC 2, age, and sex with PedsQL total score			
Term	Estimate	*SE*	*p*
Intercept	127.19	10.22	<.001
CDI 2 ineffectiveness	−0.64	0.17	.0002
CDI 2 interpersonal problems	−0.03	0.15	.82
MASC 2	−0.27	0.16	.09
Sex (female)	3.69	3.23	.26
Age	−1.30	0.62	.04

*Note.* PedsQL = Pediatric Quality of Life Inventory; MASC 2 = Multidimensional Anxiety Scale for Children, second edition; CDI 2 = Children’s Depression Inventory, second edition.

As a supplementary analysis, we investigated whether Emotional Problems, Functional Problems, or both, were the primary drivers for the relationship between the CDI 2 and PedsQL. Only the Functional Problems scale predicted PedsQL mean score (est. = −0.54, *p* = .006). All other variables were not significant (all *p*s > .05).

Given that the Functional Problems scale is composed of the Ineffectiveness and Interpersonal Problems subscales, we further investigated through supplementary analysis if either subscale was the primary driver of the correlation between Functional Problems and the PedsQL mean score. We found that the Ineffectiveness subscale score was significantly associated with PedsQL mean score (est. = −0.64, *p* = .0002), whereas the Interpersonal Problems subscale was not significantly associated (*p* > .05).

## Discussion

In studies of long COVID, mood and anxiety symptoms are commonly reported, but few studies have used validated measures (Mat Hassan et al., 2023). This study sought to elucidate prevalence rates of anxiety and depression for a clinical sample of individuals diagnosed with long COVID. When considering self-reported scores 1 *SD* from the mean and above on the MASC 2 and CDI 2, over half of patients identified having both significant anxious and significant depressive symptoms. When considering self-reported scores 2 *SD*s from the mean (considered very elevated), almost one third of patients identified having both significant anxious and significant depressive symptoms. Worldwide prevalence for children and adolescents for anxiety problems is 11.1% (95% CI [10.4–11.7]) and for depression is 5.4% (95% CI [5.0–5.9]) as of 2021 (Leeb et al., 2024). This suggests that anxious and depressive symptoms may be higher for those with long COVID than in the general population, and that many children and adolescents with long COVID experience both anxious and depressive symptoms. However, the measures used during this study do include physical symptoms of anxiety and depression that may overlap with physical symptoms of long COVID (e.g., fatigue, heart racing, shaking, dizziness, difficulties with eating, etc.), which may influence the total scores. Careful interpretation, particularly if elevations occur for Generalized Anxiety Disorder or Panic on the MASC or for Negative Mood/Physical Symptoms scale of the CDI 2 (which have more questions related to physical symptoms), and comparison with collateral information obtained from patients and families is warranted.

For depression, prevalence rates based on the CDI 2 in our sample were higher than were found by Scarselli et al. (2023); however, they also excluded participants with a prior mental health diagnosis. When excluding patients with a prior mental health diagnosis from our sample, prevalence rates for depressive symptoms were similar to Scarselli et al. For anxiety, Scarselli et al. (2023) found comparable elevations; however, given that they did not include individuals with a prior mental health diagnoses, their rates are much higher when compared to our sample. Guido et al. (2022) found a significant difference between individuals with long COVID symptoms versus individuals without long COVID symptoms for the specific anxiety subtypes of generalized anxiety, social anxiety, obsessive compulsions, and physical symptoms using the MASC 2. When looking at specific scales of the anxiety measure, our sample had more individuals with elevated scores in the domains of Generalized Anxiety and Physical Symptoms scales compared to the other scales. As mentioned previously, these two domains include questions that are more related to physical symptoms (e.g., fatigue, dizziness, heart racing, etc.). It may be that these domains are more elevated as they overlap with common physical symptoms associated with conditions of long COVID such as postural orthostatic tachycardia syndrome (Raj et al., 2018). This could, in turn, potentially artificially elevate the total score. If scores are elevated more broadly (e.g., they include domains such as separation anxiety/phobias, social anxiety, obsessive–compulsive disorder), it may be more likely that an anxiety disorder is present as these domains have fewer questions specific to physical symptoms. Similar to the discussion above regarding scores, further evaluation by a mental health professional may be useful to differentiate whether someone meets criteria for an anxiety or mood disorder in addition to the physical symptoms of long COVID, as some patients might be over-diagnosed with anxiety by solely using screening scales.

While individuals with a premorbid mood or anxiety diagnosis were more likely to have elevations on the CDI 2 and MASC 2, it is important to note that there were still individuals who had elevated scores for depressive and anxious symptoms (17% and 23%, respectively) without premorbid concerns for depression or anxiety. Importantly, about half of our sample did not have any premorbid history of depression or anxiety. Thus, there is still a significant portion of children and adolescents without a premorbid history of depression or anxiety who go on to develop new depressive and/or anxious symptoms as part of their long COVID symptoms. This outcome may be related to a negative impact on daily functioning based on onset of long COVID symptoms (Buonsenso et al., 2023). Changes in mood and/or anxiety may also be sequelae of long COVID itself (Fernández-Castañeda et al., 2022; Wong et al., 2023); however, more research in this area, particularly in pediatrics, would be needed to further understand this potential outcome.

Based on previous research (Chen et al., 2023), we investigated the relationship between depression and health-related quality of life using validated measures. Based on the cutoffs suggested by Huang et al. (2009), this cohort of patients with long COVID would have a quality of life similar to others with a “major health condition.” This is also true for the specific subscales of Physical Functioning, Emotional Functioning, and School Functioning. Social Functioning was slightly higher and would fall in the range of a “moderate health condition.” We found that higher scores on the CDI 2 were associated with lower total quality of life scores on the PedsQL. The relationship between mood and quality of life has been demonstrated in other pediatric populations with chronic medical conditions (e.g., celiac disease, Belpınar et al., 2023; diabetes, Hashim & Rahman, 2010; chronic kidney disease, Kogon et al., 2016; sickle cell disease, Sehlo & Kamfar, 2015). Our results extend prior research by clarifying which depressive symptoms are most associated with quality of life. We found that the Functional Problems scale, and specifically the Ineffectiveness subscale of the Function Problems subscale, was predictive of the total score of the PedsQL in this population. Higher scores on the Ineffectiveness subscale suggest that the individual is negatively evaluating their abilities and school performance or experiencing a decreased capacity to enjoy activities (Kovacs, 2011). As this scale relates to impact on daily functioning, it seems to align with the PedsQL. Taken together, our results suggest that while depressive symptoms may be related to an individual’s quality of life, the primary predictor of quality of life in children and adolescents with long COVID appears related to their ability to participate in daily activities (e.g., school, sports, clubs, etc.) from both physical and emotional symptoms experienced in long COVID. In clinical practice, working toward helping patients set gradual functional goals to help them get back to their daily routine (e.g., school, extra-curricular activities) may be helpful to address overall mood and quality of life. Further study would be helpful to determine if the Ineffectiveness subscale and the PedsQL are targeting the same domain, leading to a natural correlation, or if it is the negative perception of self and abilities that is truly predictive of health-related quality of life.

Our study includes a large clinical population; however, there are some limitations to the study to consider. First, this study is retrospective in nature and does not have a control group to draw direct comparisons between individuals with pediatric long COVID and individuals without long COVID. However, prior research (Lopez-Leon et al., 2022; Mat Hassan et al., 2023) suggests increased concerns for mood and anxiety in this population and this study contributes to the broader understanding of pediatric long COVID, a newer condition worldwide. Additional prospective studies and studies with control samples are warranted. Due to the fact that it is a clinical sample self-referred to a specialty clinic focused on pediatric long COVID, it is possible there is selection bias, and results may be less generalizable to individuals with milder cases of long COVID and/or those who did not seek specialty care. Finally, our screening measures capture symptoms, not clinical diagnosis of anxiety and depression.

Here we found that approximately 1 in 2.5 children and adolescents with long COVID experienced anxious symptoms, and 1 in 3 experienced depressive symptoms, yet this was not only due to a pre-existing history of mood or anxiety disorders (1 in 4 with new anxious symptoms, and 1 in 7 with new depressive symptoms). Given the evidence of increased likelihood of anxiety and depression in pediatric patients with long COVID, it is important to include assessment of these concerns for patients with long COVID (Mat Hassan et al., 2023). Also, the long-term impact of long COVID still needs to be monitored to best understand the true burden in pediatric populations (Lopez-Leon et al., 2022). The use of validated, self-reported screening measures can lead to increased identification of children with mental health concerns (Mat Hassan et al., 2023), which can then hopefully lead to more efficient connection to appropriate intervention. However, screening measures for anxiety and depression may overlap with physical symptoms of long COVID; thus, comparison with additional clinical information (e.g., clinical interview) is warranted to help determine the best treatment approach for this population.
